# Vector Research Addressing Country Control Needs

**DOI:** 10.1371/journal.pntd.0003376

**Published:** 2015-01-08

**Authors:** Yeya Tiemoko Touré, Bernadette Ramirez, Johannes Sommerfeld

**Affiliations:** 1 Retired from TDR as of 30 June, 2014; Faculté de Médecine et d′Odonto-stomatologie (FMOS), Bamako, Mali; 2 Special Programme on Research and Training in Tropical Diseases (TDR), a co-sponsored programme of UNICEF/UNDP/World Bank/WHO, based at the World Health Organization, Geneva, Switzerland; Barcelona Centre for International Health Research (CRESIB), Spain

## Introduction

Vector-borne diseases, including malaria, dengue, schistosomiasis, leishmaniasis, Chagas disease, yellow fever, lymphatic filariasis, African trypanosomiasis, and onchocerciasis, occur in more than 100 countries [1] and affect more than half of the world's population [2]. Transmitted by insect vectors, or with the involvement of intermediate or reservoir hosts, these are among the most neglected tropical diseases. Vector-borne diseases account for 17% of the estimated global burden of all infectious diseases [2]–[4].

Effective prevention strategies can reverse this trend of high disease burden. Vector control as a method to reduce or interrupt transmission [4] is a key component of such strategies. New and improved vector control tools and strategies are needed, and research must more directly address countries' needs to improve the effectiveness of disease control [5].

This article compares TDR strategic emphases [6] and basic research on vectors before 2007 against the current research in support of control interventions (2008 to date), documenting the changes in research output, training, and practical application, from basic science to more directly addressing country needs for scientific evidence and improved control tools and strategies.

## More Than Ten Years of Support to Investigator-Driven Basic Research

TDR supported investigator-driven vector research in molecular entomology from 1994 to 2007. Prior to 2007, a research strategy was developed for each of the ten diseases then in the TDR portfolio (Chagas disease, dengue, human African trypanosomiasis (HAT), leishmaniasis, leprosy, lymphatic filariasis, malaria, onchocerciasis, schistosomiasis, and tuberculosis) based on a seven-step analysis [6]. The vector research focused on the “new and basic knowledge” research area and was implemented through investigator-driven research following competitive calls for applications, with the proposals and progress reports reviewed by an external scientific review committee.

The TDR Molecular Entomology Committee (BCV) was created in 1994 following the Tucson, Arizona, (United States) meeting of 1991 on "Prospects for malaria control by genetic manipulation of its vectors" [7]. The main objective for the 15-year program (1995–2010) was to develop tools for genetic modification of mosquitoes, identify genes to make mosquitoes unable to transmit the parasites, develop methods for spreading the genes in wild mosquito populations, and field test the genetic control methods.

The research activities were focused on molecular biology, genomics, and genetic modification of vectors of malaria, dengue, and HAT. The accompanying capacity building activities included courses on the Biology of Disease Vectors (BDV, from 1994–2007), and bioinformatics and functional genomics applied to insect vectors (2004–2011).

These activities produced important achievements, including developing genetic modification methods for malaria vectors [8], [9] and sequencing the genomes of the malaria vector *Anopheles gambiae* in 2002 [10] and of the HAT vector *Glossina morsitans* in 2014 [11]. In addition, they generated new basic knowledge on insect biology and genetics and supported the training of 180 researchers in bioinformatics and functional genomics [12] and 100 researchers in the BDV courses [13]. The potential for this new knowledge to help develop new and innovative vector control tools and strategies was highlighted by Morel et al. in 2002 [14] and Touré et al. in 2004 [15].

In 2006, TDR commissioned an evaluation of the vector research activities undertaken between 1994 and 2005 to inform the implementation of the 2008–2017 strategy. Of the 104 research projects funded, 72% were from US and European investigators and 28% from 11 disease-endemic countries in Africa, Asia, and Latin America. In addition, more than 95% of the funded research projects were about malaria vectors. The programme trained 380 scientists, with 26.3% trained within the research projects and 73.7% as part of 26 specific training projects. Most of the trainees were from disease-endemic countries. The research activities generated 341 publications (an average of more than three publications per project).

Overall, the programme was evaluated positively, showing mainly laboratory-based research driven by northern investigators who had the expertise and the facilities and who published a large amount of scientific information. It also contributed significantly to building research capacity in disease-endemic countries. However, despite the programme's importance, the scientific information generated would clearly need time to find practical applications for disease-endemic countries' vector control programmes.

The evaluation recommended focusing on a few selected topics going forward, providing substantial funding to fewer projects and ensuring that training remains a key component of the activities.

## From Investigator-Driven Research to Research Addressing Countries' Needs

During the same time in 2006, an external review of TDR was undertaken that led to the development of a new strategic plan with a change in direction that would have an impact on vector research priorities.

The 2006 TDR external review concluded, “TDR's focus should be on the very neglected diseases, and even more so on the health needs of the most needy **populations**” [16]. It further highlighted the need for a strong link between research and control in relation to the scaling up of interventions, policies, and the use of tools [16].

A ten-year vision and strategy (2008–2017) followed in December 2006 [17]. TDR's vision in this strategy was to foster “An effective global research effort on infectious diseases of poverty, in which disease-endemic countries play a pivotal role.” In order to achieve this, TDR focused support on innovative research for disease control priorities and significantly increased support for implementation research.

Vector research took a more holistic approach that continues today. The main objective is to develop and evaluate new and improved vector and vector-borne disease control methods and strategies in the context of social, environmental, and climate change. The research also explores optimal ways to engage communities in the delivery and scale-up of control interventions.

The current vector research programme (2008–2017) had supported 24 multi-country commissioned projects selected through competitive call for applications. The research topics were identified by TDR following a review of control and research needs during informal consultations in scientific working group meetings. The strategic framework and operational approach for TDR vector research was defined during a broad stakeholder and expert consultation meeting in April 2007. The recommendations from the consultation were used to develop a TDR vector research work plan with clear expected outputs and outcomes and their expected indicators for each strategic objective. The work plan (including its planned budget) was reviewed by the TDR Scientific and Technical Advisory Committee (STAC) before its approval by the Joint Coordinating Board (JCB). Examples of topics included “development and evaluation of improved tsetse control methods and strategies,” “development and evaluation of improved targeted and integrated dengue vector control methods and strategies,” “development and evaluation of improved methods and strategies for packaging integrated malaria vector control approaches,” and “development and evaluation of strategies for complementary or alternative Chagas disease vector control measures.”

Following the approval of the work plan, calls for applications for commissioned research were issued and posted on TDR website for about two months. The selection of proposals and review of progress reports were made by an independent scientific review committee appointed by the TDR director. The selection of the projects was based on a ranking using criteria of scientific merit, relevance to TDR work plan, and feasibility under the available funding and working conditions. The number of projects to be funded was determined by the budget available for the activity.

The recommendations from the review committee were submitted to the TDR director for final approval of the funding of the projects. The projects approved for funding were officially announced with summary information posted on TDR website. All the applicants received official decision letters with the detailed comments from the reviewers justifying the decision for approval or rejection of the application.

The funded projects included ten projects on vector control methods and strategies in Africa, Asia, and Latin America; five on environmental and climate change impact on vector-borne diseases (VBDs) in Africa; eight on community-based dengue and Chagas disease vector control interventions in Latin America and the Caribbean; and one on community-based dengue vector control interventions in six Asian countries. For all the research projects, it was mandatory for researchers to involve the communities, control services, and decision makers right at the beginning in the planning and implementation process.

The research activities have already had positive effects in providing scientific evidence and improved vector control tools and strategies that helped to improve control interventions. The achievements include the standardization and optimization of trapping methods for six HAT vectors across Africa in nine countries (Angola, Côte d′Ivoire, Burkina Faso, Democratic Republic of the Congo, Kenya, Malawi, Sudan, Tanzania, and Uganda) [18]–[20], the identification of gaps and weaknesses in ongoing malaria control interventions in three African countries (Cameroon, Kenya, and Mali), the examination of sources of re-infestation of houses by triatomine bugs, and the analysis of the most suitable insecticides for house spraying for Chagas disease control in seven Latin American countries (Argentina, Bolivia, Brazil, Colombia, Mexico, Panama, and Paraguay) [21].

In addition, the achievements included a proof of principle of efficacy in reducing vector densities [22] through dengue vector control interventions in Asia (Thailand and Viet Nam) and Latin America (Guatemala and Brazil), combining the use of insecticide-treated materials (door/window curtains and water jar covers) with targeting the most productive *Aedes* mosquito larval breeding sites and biological control method (using larvivorous fish). This proof of principle was also demonstrated through ecological, biological, and social studies conducted between 2006 and 2011 in urban and semi-urban areas in Asia (India, Myanmar, Sri Lanka, Indonesia, Philippines, and Thailand) that helped develop community-based intervention [23].

Moreover, the achievements included the identification of key factors associated with vector breeding and development as a basis for improving targeted intervention strategies [24]. The results helped in designing multicentre intervention studies in five Latin American countries (Mexico, Colombia, Ecuador, Brazil, and Uruguay) that were further evaluated and showed the benefit of targeted vector management in reducing vector abundance.

The 19 research projects funded from 2008–2013 generated 62 publications, with an average of over three publications per project; the five projects on environmental and climate change impact on VBDs that started in 2013 are still to be published.

Examples of detailed information about the projects, diseases, countries involved, project cost, and list of publications are accessible in the annual reports (2008–2011) for TDR innovative vector control interventions under “Annexes: list of publications; funded projects” at http://whqlibdoc.who.int/hq/2010/TDR_BL5.10_eng.pdf and http://www.who.int/tdr/publications/documents/bl5-annual-report-2008.pdf?ua=1.

In addition to the vector research activities referred to above that were conducted under the innovative vector control interventions research portfolio, TDR provided support to countries for vector control and reduction of transmission for the elimination of visceral leishmaniasis (VL) in the Indian subcontinent (Bangladesh, India, and Nepal). The VL vector research activities conducted under the implementation research portfolio helped provide to countries monitoring and evaluation guidelines for VL vector control interventions [25]–[27].

## Effects of Shifts in Vector Research Strategy

TDR-supported research demonstrated a relatively acceptable cost-benefit by leveraging about three to four times its funding equivalent through contributions by countries and other partners to the projects. However, the uptake of research findings from TDR-funded projects by health systems of developing countries has been insufficient. This is in part due to a lack of appropriate translation and communication of results.

The current shift in TDR research strategy is intended to improve this situation through more addressing of country needs for scientific evidence and improved control tools and strategies, as well as involving communities, control services, and decision makers in the research process for better communication and uptake of research findings.

Overall, the shift was intended to make the research outputs more relevant and useful for problem-solving and practical applications in control programmes, and as indicated below, it is on track for this purpose.

Before 2007, there were 104 single-country basic research projects funded over ten years; 72% of these were from northern country investigators. After 2007, there were 24 multicountry implementation research projects funded in five years, with 79% from disease-endemic country investigators. This shows a reversal of the pattern for leadership, with more southern investigators driving the funded projects, and of the type of projects funded, which shifted from basic research to implementation research and to fewer but larger, well-funded, multicountry projects. An indication of the practical aspect of the implementation research activities is that the results are already used for practice changes. For example, the national malaria control programme in Mali, in agreement with the donor agencies, changed the insecticide they were using for indoor residual spraying (IRS) of houses from lambdacyhalothrin to carbamate. This decision was based on poor IRS efficacy results with lambdacyhalothrin detected by the researchers in the malaria vector control programmes. Another example comes from the TDR dengue vector control research activity in six countries in Asia, which resulted at community level in local groups being united around broad interests in environmental hygiene and sanitation (including vector control).

The shift in TDR vector research strategy can also be seen by looking at which countries were involved. Before 2007, there were 11 disease-endemic countries (Africa: four, Asia: three, and Latin America: four) involved in 28% of the research. After 2007, 84% of the 62 countries involved in the research projects were from disease-endemic countries (Africa: 21, Asia: 14, and Latin America: 17) and they accounted for 79% of the projects. This shows a shift towards the disease-endemic countries becoming more involved in the research activities.

The disease focus also changed over this period. Before 2007, 95% of projects were devoted to malaria. After 2007, there was a more even distribution among the diseases studied (Chagas disease: five, malaria: two, dengue: eight, HAT: four, leishmaniasis: three, and multidisease projects: five).

These changes did not seem to negatively affect the average publication output. Before 2007, there was an average of 3.28 publications per project (341 publications for 104 projects over ten years), and after 2007, there was an average of 3.26 publications (62 publications for 19 projects over five years). The total funds allocated for research per year was US$567,190 before 2007 and 2 million after 2007. Although the funds allocated per year during the second phase were much larger than those of the first phase, it is important to note that for most of the projects during the first phase, researchers from developed countries had complementary activities funded from other funding sources. In addition, the implementation research projects during the second phase took much longer to deliver and to be ready for publication, and they have more public health value.

In terms of operating procedures, TDR moved from having the investigators propose the research topics unguided to identifying research priorities in consultation with stakeholders, national disease control programs, researchers, donor agencies, and worldwide experts before inviting investigators to address the identified priorities through competitive calls for applications for commissioned research.

The current focus for TDR vector research is on implementation research to optimize control interventions. This research takes into account the complexity of the environment, including developmental activities (agriculture, irrigation, dams, deforestation) and the need for integrated methods, transdisciplinary approach to disease control, and better coordination and cooperation for disease research and control. The expected result is locally adapted solutions to countries' control problems.

The current research provides scientific evidence and improved tools and strategies for better planning and implementation of control interventions with the direct involvement of communities, disease control services, and decision makers in the research process. Consequently, it allows better communication and uptake of research results and sustainability of the efforts and gains. This approach is particularly relevant to the complex research of developing adaptation strategies to the impact of climate change on vector-borne diseases.

## Conclusion

TDR-supported vector research under the new strategic direction has already contributed to improved control interventions by optimizing and standardizing methods and providing scientific evidence for better planning and implementation of the targeted and integrated interventions in several disease-endemic countries. It has particularly improved collaboration between researchers and disease control personnel, thereby facilitating the uptake of results. Moreover, it has increased the number of research contributions from scientists living in the countries where the problems occur.

The integrated, multidisciplinary approach, driven by country needs for scientific evidence and improved control tools and strategies and involving more local researchers and community members, has been shown to address country needs faster and with good results. There is still much to be done, but we believe this is an approach that can increase the pace of progress against a range of important diseases.
